# Associations of hippocampal volumes, brain hypometabolism, and plasma NfL with amyloid, tau, and cognitive decline

**DOI:** 10.1002/alz.70005

**Published:** 2025-02-24

**Authors:** Feng‐Feng Pan, Qi Huang, Chu‐Chung Huang, Yao Lu, Liang Cui, Lin Huang, Yihui Guan, Fang Xie, Qi‐Hao Guo

**Affiliations:** ^1^ Department of Gerontology Shanghai Sixth People's Hospital Affiliated to Shanghai Jiao Tong University School of Medicine Shanghai China; ^2^ PET Center Huashan Hospital Fudan University Shanghai China; ^3^ Shanghai Key Laboratory of Brain Functional Genomics (Ministry of Education) Affiliated Mental Health Center (ECNU) School of Psychology and Cognitive Science East China Normal University Shanghai China

**Keywords:** 18F‐fluorodeoxyglucose positron emission tomography, Alzheimer's disease, amyloid positron emission tomography, hippocampal volume, plasma neurofilament light chain, plasma phosphorylated tau181

## Abstract

**INTRODUCTION:**

Various indicators of neurodegeneration (N) are used in the assessment of neuronal injury in Alzheimer's disease (AD). The heterogeneity of such indicators is less clear.

**METHODS:**

A total of 416 individuals with different cognitive statuses were recruited for this study. Differential associations of hippocampal volume (HV), 18F‐fluorodeoxyglucose positron emission tomography (FDG PET) standardized uptake value ratios (SUVRs), and plasma neurofilament light chain (NfL) levels with amyloid beta (Aβ)–tau pathology and cognitive impairment were examined.

**RESULTS:**

HV decreased early during the high Aβ burden but tau‐negative stage. FDG PET SUVRs and plasma NfL levels notably changed at tau‐positive stages. HV and plasma NfL correlated with cognitive scores in the early to middle stages, while FDG PET SUVRs aligned with cognitive decline from the middle to late stages. Hippocampal atrophy and inferior parietal hypometabolism increased the risk of cognitive impairment in A+T+, while adding NfL+ had no additional impact within the distinct A/T groups.

**DISCUSSION:**

Different indicators of N have varying relationships to AD pathology and cognitive impairment.

**Highlights:**

- Hippocampal atrophy emerges early with a high amyloid beta burden and exacerbates during the tau‐positive phase.
- Brain hypometabolism and elevated plasma neurofilament light chain (NfL) levels appear mainly in tau‐positive stages.
- Hippocampal volume and plasma NfL levels correlate with cognitive decline in the early to middle stages, while 18F‐fluorodeoxyglucose positron emission tomography standardized uptake value ratios in the middle to late stages.
- Hippocampal atrophy and inferior parietal hypometabolism raise the risk of cognitive impairment in amyloid/tau–positive individuals while adding NfL+ shows no additional effect.

## 1 BACKGROUND

Alzheimer's disease (AD) is the most common cause of dementia in the elderly and has emerged as a significant public health concern.^1^ For the early and accurate diagnosis of AD, the National Institute on Aging and the Alzheimer's Association (NIA‐AA) has presented a biological definition of AD known as AT(N).^2^ Compared to the core pathological changes of amyloid beta (Aβ; A) and tau (T), the additional indicator of neurodegeneration (N) is not AD specific but more closely related to cognitive impairment.^3^ In the recently revised diagnostic and staging criteria for AD, the indicators of N are mainly observed from anatomic magnetic resonance imaging (MRI), 18F‐fluorodeoxyglucose positron emission tomography (FDG PET) scans, and cerebrospinal fluid (CSF) or plasma‐based neurofilament light chain (NfL).^4^ Previous studies have suggested relatively characteristic changes of these indicators in AD patients, such as decreased hippocampal volume (HV), AD‐vulnerable cortical thinning on anatomic magnetic resonance imaging (MRI), and hypometabolism in temporoparietal association areas, including posterior cingulate cortex on FDG PET.^5^, ^6^ However, apparent inconsistencies were observed when using these indicators to define N positivity (N+) in AD^7^, ^8^ and in the correlation between these indicators and cognitive decline.^9^, ^10^ The discrepancies among these indicators of N may be attributed to their distinct characteristics. Specifically, anatomic MRI captures cumulative neuropil loss and brain atrophy, FDG PET reveals neuronal functional impairment, and fluid biomarkers indicate the severity of neuronal injury at a specific point in time.^11^, ^12^, ^13^ Beyond this, the inconsistent results may also be attributed to insufficient consideration of the impacts of amyloid–tau pathology, as well as the differential associations between these indicators and cognitive decline. To advance our understanding of the disparate findings among neurodegenerative biomarkers and to establish a foundation for clinical application, it is essential to examine the associations between these biomarkers and amyloid–tau pathology, as well as their correlation with cognitive decline throughout the progression of the disease.

In this study, we primarily aimed to identify the differences in the associations between various indicators of N and the core AD biomarkers of Aβ and tau. Meanwhile, the study seeks to assess the disparities in the relationships between these indicators of N and cognitive decline. According to the AT(N) categorization in the revised criteria for the diagnosis and staging of AD,^4^ 18F‐florbetapir (AV45) PET was used to evaluate Aβ deposition, plasma phosphorylated tau (p‐tau)181 levels were measured to assess tau pathology, and N was characterized by decreased hippocampal volume (HV) on anatomical MRI, reduced brain metabolism on FDG PET, and elevated plasma NfL levels.

## 2 METHODS

### 2.1 Study participants

Data were obtained from the Chinese Preclinical Alzheimer's Disease Study (C‐PAS), initiated in 2019 and designed to establish a large‐scale research cohort in a Chinese population for earlier and accurate diagnosis and prognosis of AD.^14^ For the present study, we used data and samples collected from March 30, 2019, to September 8, 2021. All participants underwent simultaneous assessments of plasma p‐tau181 and NfL using single molecule array (Simoa), HV via anatomical MRI, and both AV45 PET and FDG PET imaging. A battery of standardized neuropsychological tests were performed in our study participants, as we previously reported, in which the Chinese version of Addenbrooke's Cognitive Examination III (ACE‐III‐CV) scale was used to assess global cognitive function.^15^ Individuals with any of the following conditions were excluded from this study: (1) neurologic diseases other than AD, such as cerebral infarction, subdural hematomas, hydrocephalus, intracranial tumors, intracranial infections, and head trauma; (2) psychiatric disorders or systemic illness which may be adversely affecting cognitive function, such as evident abnormalities in folic acid, vitamin B12, and thyroid function. Participants with or without cognitive complaints but who performed normally on the standardized neuropsychological tests were defined as cognitively normal (CN). Those with self‐reported or informant‐identified cognitive decline and who had objective cognitive impairment verified via standardized neuropsychological tests were defined as cognitively impaired (CI). Written informed consent was obtained from all the participants or their caregivers. The ethics committee of Shanghai Sixth People's Hospital, affiliated with Shanghai Jiao Tong University School of Medicine, reviewed and approved this study.

### 2.2 Aβ, tau assessments, and A/T classification

Brain Aβ deposition was assessed by AV45 PET scans using a PET/computed tomography system (Biography 64 PET/CT, Siemens) in the PET Center, Huashan Hospital, Fudan University. Scans were performed 50 minutes after the intravenous injection of 7.4 MBq/kg (0.2 mCi/kg) 18F‐florbetapir and lasted for 20 minutes. PET images were reconstructed by a filtered back projection algorithm with corrections for decay, normalization, dead time, photon attenuation, scatter, and random coincidences. Images were coregistered to the individual anatomic MRI, and partial volume error correction (PVC) was performed using the Muller–Gartner method,^16^ followed by spatial normalization in the Montreal Neurological Institute (MNI) template and Gaussian smoothing. Subjects were classified as Aβ positive (A+) by visual rating according to the guidelines previously reported.^17^ Brain Aβ burden was measured via the standardized uptake value ratios (SUVR) for the global cortical region, which was calculated by weighted averaging of the region of interest (ROI). The whole cerebellum was used as the reference region. AD‐related tau pathology was assessed by the levels of plasma p‐tau181, which was measured via P‐Tau 181 Assay Kit V2 (Lot 502923) on the Quanterix Simoa HD‐1 platform.^18^ Sample collection, preprocessing, and detection procedures were carried out as we previously reported.^19^ Tau positivity (T+) was determined based on the cut point 2.914 pg/mL with the highest Youden index to differentiate CI individuals with brain Aβ positivity from CN individuals who were brain Aβ negative. All the participants were classified into four subgroups according to their statuses of Aβ and tau (A−T−, A−T+, A+T−, and A+T+). In the subgroup of A+T−, individuals with AV45 SUVR values in the lower and middle tertiles were classified as A+_Low_T− (AV45 PET SUVR ≤ 0.925), while those with AV45 SUVR values in the upper tertile were classified as A+_High_T− (AV45 PET SUVR > 0.925). Similarly, in the subgroup of A+T+, individuals were further classified into A+T+_Low_ (P‐tau181 ≤ 4.529 pg/mL) and A+T+_High_ (P‐tau181 > 4.529 pg/mL) based on the tertiles of plasma p‐tau181 levels. Consequently, excluding the A−T+ subgroup, we categorized the participants into A−T−, A+_Low_T−, A+_High_T−, A+T_Low_+, and A+T_High_+, in alignment with the classical pathological progression of AD.

**RESEARCH IN CONTEXT**

1. **Systematic review**: The authors used PubMed to conduct their literature search. While various indicators of neurodegeneration (N) are used to assess neuronal injury in Alzheimer's disease (AD), noticeable discrepancies exist among the evaluation results of different indicators. Studies that simultaneously analyze different biomarkers in relation to AD pathology and cognitive impairment are lacking.
2. **Interpretation**: In this cross‐sectional study, we observed that hippocampal volume, cerebral metabolism, and plasma neurofilament light chain levels demonstrate distinct characteristics in response to increasing amyloid beta (A) and tau (T) pathology. The correlations between these biomarkers and cognitive function varied at different degrees of cognitive decline. Additionally, neurodegenerative markers show different correlations with cognitive impairment across various A/T groups.
3. **Future directions**: Our findings support the utility of distinct indicators of N in diverse clinical contexts. Further studies are needed to precisely determine the role of these biomarkers and their implementation for the assessment of neuronal injury in clinical settings.

### 2.3 Indicators of N

Brain MRI images were acquired using a 3.0 T MRI scanner (SIEMENS MAGNETOM Prisma 3.0T, Siemens) at the Department of Radiology, Shanghai Sixth People's Hospital. 3D T1‐weighted MRI images were processed using the automated analysis pipeline of FreeSurfer v6.0 (http://freesurfer.net/). The preprocessing steps included format conversion, motion correction, removal of non‐brain tissue, affine transformation to the Talairach atlas, intensity normalization, gray–white matter segmentation, automatic topological correction, and surface deformation. The left and right hippocampus volumes (HV‐L, HV‐R) were calculated by dividing the total HV by the estimated total intracranial volume and multiplying by 1000. FDG PET was conducted in the PET Center, Huashan Hospital, Fudan University. Scans were performed 50 minutes after the intravenous injection of 5.55 MBq/kg (0.15 mCi/kg) 18F‐fluoro‐2‐deoxyglucose and lasted for 10 minutes. ROIs, including the left and right inferior parietal (IP‐L, IP‐R), middle temporal (MT‐L, MT‐R), and posterior cingulate (PC‐L, PC‐R) gyri, were used to evaluate the metabolic activity of brain tissue. SUVRs were calculated in each ROI using the whole cerebellum as a reference. Plasma levels of NfL were measured via NF‐light Assay Kit (Lot 202700) on the Quanterix Simoa HD‐1 platform.^18^

### 2.4 Statistical analyses

Categorical variables were expressed as numbers (percentages) and analyzed using the chi‐squared test. Continuous variables were expressed as means  ±  standard deviation. Independent samples *t* tests were used for two‐group comparison. Multi‐group comparisons were analyzed using the analysis of covariance (ANCOVA) with post hoc Bonferroni correction. Sex, age, education years, and apolipoprotein E (*APOE*) ε4 genotype were used as covariates. A heat map of Pearson correlation was constructed to evaluate the correlation between various indicators of N across different cognitive and amyloid statuses, with multiple testing corrections applied using the false discovery rate (FDR) method. Generalized linear models (GLMs) were used to evaluate the associations between demographic variables and amyloid–tau pathology with various indicators of N. Mediation analyses were further conducted using SPSS PROCESS macro (Model 4) to investigate the associations between multiple indicators of N and amyloid–tau pathology. The global AV45 PET SUVR was modeled as the associated variable, plasma p‐tau181 level was included as the mediating variable, and each of the indicators of N was assessed separately as the outcome variable. Covariates used in the models included sex, age, education years, and *APOE* ε4 genotype. All paths are reported as standardized regression coefficients, and the confidence intervals for the direct and indirect effects were estimated with 5000 bootstrap samples. AV45 PET SUVR and plasma p‐tau181 levels were log‐transformed both in the GLM and mediation models. Segmental linear regression was used to compare the variation characteristics of various indicators of N in relation to changes in global cognitive decline. Indicators of N were *z* scored before analysis. Logistic regression analyses were carried out with cognitive impairment as the outcome and N+ in different A/T statuses as the primary exposures. A two‐sided *P* value < 0.05 was considered statistically significant. Data analyses were performed with IBM SPSS Statistics 26 and GraphPad Prism 9.0 (GraphPad Software, Inc).

## 3 RESULTS

### 3.1 Participant characteristics

Among the 416 participants included in this study, the mean age was 64.93 years, 37.7% were male, 240 were classified as CN, and 176 were classified as CI (Table 1). In comparison, individuals with CI exhibited significantly fewer years of education, lower performance on the ACE‐III‐CV, and higher scores on the Hamilton Depression Scale (HAMD). Additionally, individuals with CI had higher proportions of *APOE* ε4+ and AV45 PET+, as well as elevated levels of AV45 PET SUVR, plasma p‐tau181, plasma NfL, and reduced HVs and FDG PET SUVRs. No significant difference in sex, age, Hamilton Anxiety Scale (HAMA), and Activities of Daily Living (ADL) scores was found between the individuals with CN and CI.

**TABLE 1:** Characteristics of the study sample.

Index	ALL (*n* = 416)	CN (*n* = 240)	CI (*n* = 176)	t/x2 (*P* value)
Sex (Male, *n* [%])	157 (37.7%)	87 (36.3%)	70 (39.8%)	0.536 (0.464)
Age (years)	64.93 ± 7.57	64.38 ± 7.89	65.68 ± 7.05	−1.739 (0.083)
Education (years)	11.45 ± 3.78	12.51 ± 3.29	10.01 ± 3.93	6.989 (< 0.001)
*APOE* ε4 + (*n* [%])	106 (25.5%)	43 (17.9%)	63 (35.8%)	17.093 (< 0.001)
ACE‐III‐CV	73.09 ± 15.49	81.35 ± 7.63	61.77 ± 16.36	16.172 (< 0.001)
ADL	21.26 ± 10.01	20.96 ± 12.53	21.66 ± 4.80	−0.692 (0.490)
HAMD	4.08 ± 4.27	3.61 ± 3.84	4.77 ± 4.78	−2.388 (0.018)
HAMA	5.74 ± 5.63	5.62 ± 5.55	5.94 ± 5.79	−0.503 (0.616)
AV45 PET + (*n* [%])	175 (42.1%)	76 (31.7%)	99 (56.3%)	25.179 (< 0.001)
AV45 PET SUVR	0.90 ± 0.19	0.87 ± 0.16	0.95 ± 0.22	−4.262 (< 0.001)
Plasma p‐tau181 (pg/mL)	2.27 ± 1.42	1.93 ± 1.04	2.73 ± 1.72	−5.912 (< 0.001)
***Anatomic MRI (mm^3^)***				
Hippocampal volume (L)	3153 ± 408	3279 ± 307	2983 ± 465	7.816 (< 0.001)
Hippocampal volume (R)	3306 ± 429	3428 ± 320	3139 ± 498	7.191 (< 0.001)
***FDG PET SUVR***				
Inferior parietal (L)	1.26 ± 0.16	1.29 ± 0.13	1.23 ± 0.19	3.308 (0.001)
Inferior parietal (R)	1.08 ± 0.16	1.10 ± 0.14	1.06 ± 0.18	2.706 (0.007)
Middle temporal (L)	1.36 ± 0.10	1.37 ± 0.08	1.34 ± 0.13	3.478 (0.001)
Middle temporal (R)	1.21 ± 0.11	1.23 ± 0.09	1.19 ± 0.14	3.594 (< 0.001)
Posterior cingulate (L)	1.51 ± 0.20	1.54 ± 0.17	1.46 ± 0.23	3.920 (< 0.001)
Posterior cingulate (R)	0.90 ± 0.16	0.92 ± 0.13	0.87 ± 0.19	2.906 (0.004)
***Plasma NfL (pg/mL)***	16.85 ± 9.62	14.92 ± 8.11	19.49 ± 10.84	−4.923 (< 0.001)

*Note*: Data are shown as means  ±  standard deviation or *n* (%). Group comparisons were performed using the independent samples *t*‐test or Chi‐squared test based on the data type.

Abbreviations: ACE‐III‐CV, Chinese version of Addenbrooke's cognitive examination III; AD, Alzheimer's disease; ADL, Activities of Daily Living; *APOE*, apolipoprotein E; AV45, 18F‐florbetapir; Aβ, amyloid beta; CI, cognitively impaired; CN, cognitively normal; FDG, fluorodeoxyglucose; HAMA, Hamilton Anxiety Scale; HAMD, Hamilton Depression Scale; HV, hippocampal volume; MRI, magnetic resonance imaging; NfL, neurofilament light chain; NPI, Neuropsychiatric Inventory; PET, positron emission tomography; p‐tau, phosphorylated tau; SUVR, standardized uptake value ratio.

### 3.2 Correlation between various indicators of N

**FIGURE 1:**
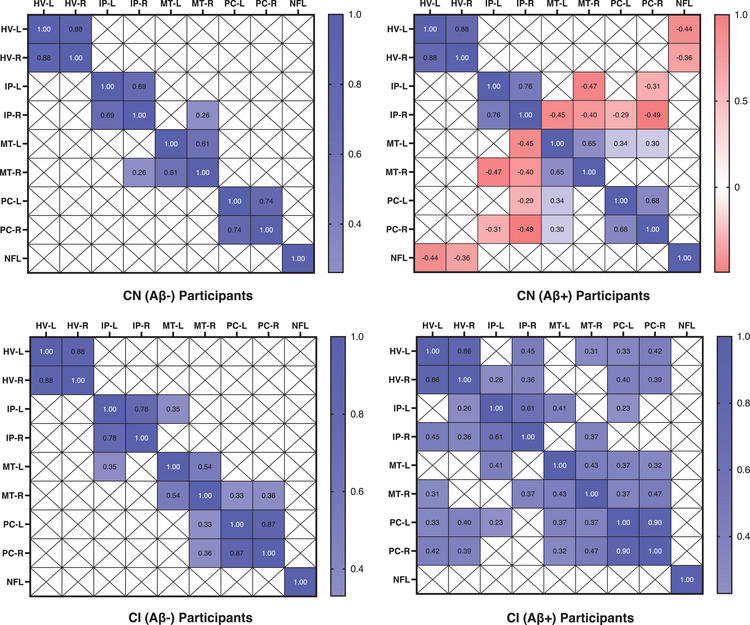
Correlation heat map for various indicators of neurodegeneration. All the presented Pearson correlation coefficients were statistically significant (false discovery rate–corrected *P* values < 0.05). Aβ, amyloid beta; CI, cognitively impaired; CN, cognitively normal; FDG, fluorodeoxyglucose; HV, hippocampal volume (magnetic resonance imaging); IP, inferior parietal (FDG PET); L, left; MT, middle temporal (FDG PET); NfL, neurofilament light chain (plasma); PC, posterior cingulate (FDG PET); PET, positron emission tomography; R, right

The heat map of correlation between various indicators of N is presented in Figure 1. In the participants who were brain Aβ negative, no significant correlation was observed among HVs, FDG PET SUVRs, and plasma NfL levels. In participants who were CN (Aβ+), significant negative correlations were observed between the volumes of the left and right hippocampus and the levels of plasma NfL (*r* = −0.44 and −0.36, respectively). HVs and plasma NfL levels showed no significant correlation with FDG PET SUVRs. In participants with CI (Aβ+), HVs exhibited significant positive correlations with FDG PET SUVRs (*r* = 0.26–0.46), except for the correlations among HV‐L and IP‐L and MT‐L, as well as among HV‐R and MT‐L and MT‐R gyri. HVs and FDG PET SUVRs showed no significant correlation with plasma NfL levels.

### 3.3 Associations between indicators of N and amyloid–tau pathology

**FIGURE 2:**
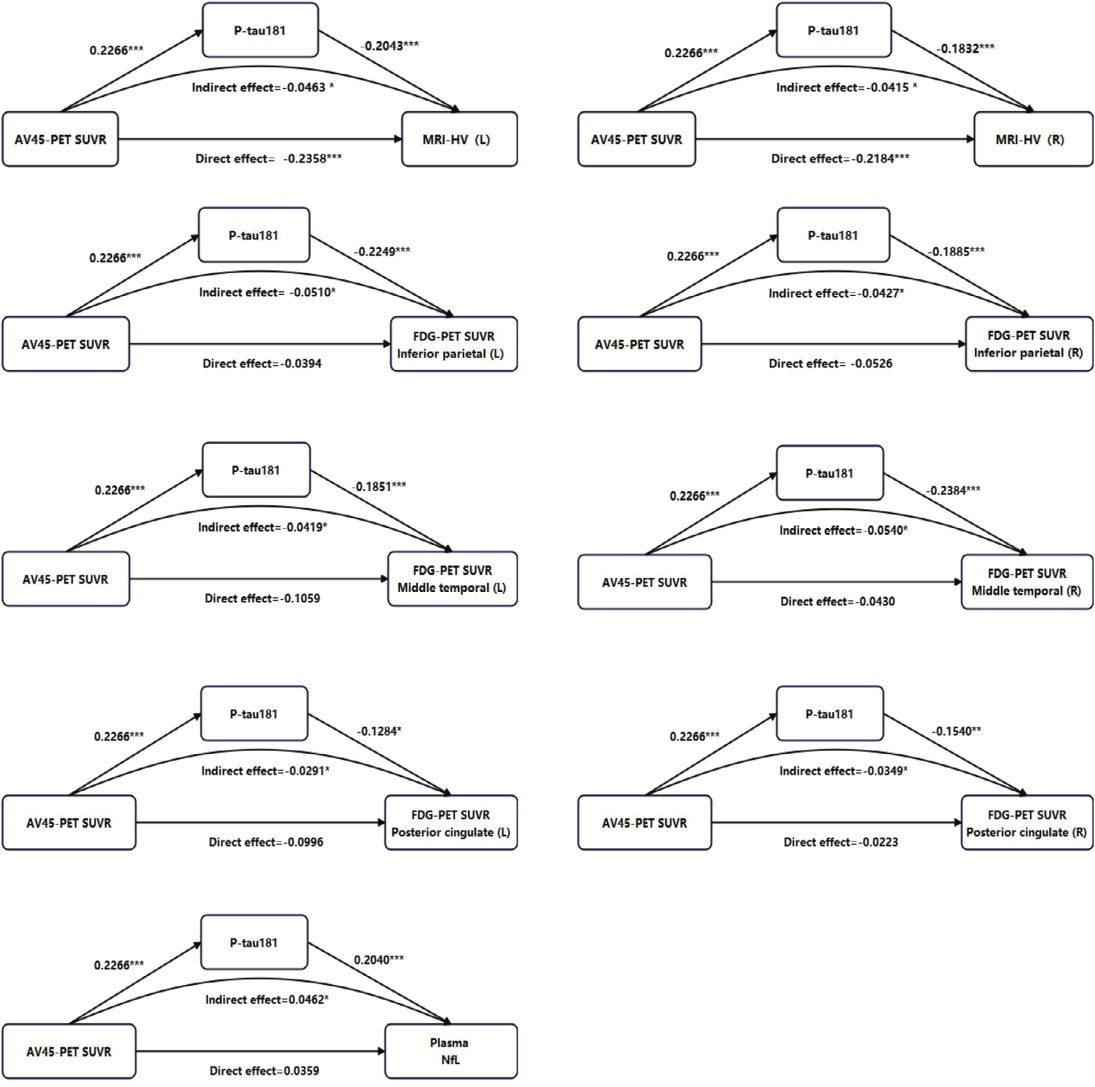
Mediating effects of plasma p‐tau181 on various indicators of neurodegeneration. Standardized coefficients are obtained from meditation analysis with AV45 PET SUVR as independent variable, plasma p‐tau181 levels as mediator variable, and sex, age, education years, and *APOE* ε4 genotype as covariates. Indicators of neurodegeneration are *z* scored before analysis. AV45 PET SUVR and plasma p‐tau181 levels are log‐transformed. *P < 0.05, **P < 0.01, ***P < 0.001. *APOE*, apolipoprotein E; AV45, 18F‐florbetapir; FDG, fluorodeoxyglucose; HV, hippocampal volume; L, left; MRI, magnetic resonance imaging; NfL, neurofilament light chain; PET, positron emission tomography; p‐tau, phosphorylated tau; R, right; SUVR, standardized uptake value ratios.

Multivariate analyses using GLMs were conducted to evaluate the effects of global AV45 PET SUVR and plasma p‐tau181 levels on various indicators of N (Table 2). After adjusting for sex, age, education years, and *APOE* ε4 genotype, an increase in AV45 PET SUVR was significantly correlated with reductions in both HV‐L and HV‐R (*β* = −3.048 and −2.827, respectively). In contrast, elevated plasma p‐tau181 levels were associated not only with decreased HV‐L and HV‐R (*β* = −0.766 and −0.688, respectively) but also with reduced FDG PET SUVRs in the IP‐L, IP‐R, MT‐L, MT‐R, PC‐L, PC‐R gyri (*β* = −0.835, −0.701, −0.694, −0.862, −0.473, and −0.573, respectively), as well as with increased plasma NfL levels (*β* = 0.766). Mediation models were further conducted to investigate the associations between multiple indicators of N and amyloid–tau pathology. As shown in Figure 2, global AV45 PET SUVR exhibited a significantly positive effect on plasma p‐tau181 (standardized coefficient = 0.2266). Plasma p‐tau181 demonstrated significantly negative effects on HV‐L and HV‐R (standardized coefficients = −0.2043 and −0.1832, respectively), FDG PET SUVRs in IP‐L, IP‐R, MT‐L, MT‐R, PC‐L, and PC‐R gyri (standardized coefficients = −0.2249, −0.1885, −0.1851, −0.2384, −0.1284, and −0.1540, respectively), as well as positive effects on the plasma NfL level (standardized coefficients = 0.2040). In addition, AV45 PET SUVR exhibited a direct negative effect on the HV‐L and HV‐R (standardized coefficient = −0.2358 and ‐0.2184, respectively), whereas no direct effect was detected on FDG PET SUVRs or plasma NfL level.

**TABLE 2:** Multivariable associations between demographic factors and amyloid–tau with different indicators of N.

Index	Anatomic MRI		FDG PET SUVR						Plasma
	HV (L)	HV (R)	Inferior parietal (L)	Inferior parietal (R)	Middle temporal (L)	Middle temporal (R)	Posterior cingulate (L)	Posterior cingulate (R)	NfL
(Intercept)	1.269^**^	0.890^*^	−1.416^**^	0.009	−0.665	0.319	−0.899	−0.705	−3.100^***^
Sex (male)	0.275^**^	0.268^**^	0.031	−0.028	−0.034	−0.307^**^	−0.099	−0.204^*^	0.085
Age (years)	−0.031^***^	−0.026^***^	0.021^**^	0.000	0.012	0.000	0.010	0.008	0.046^***^
Education (years)	0.033^**^	0.042^***^	0.010	0.004	0.011	−0.001	0.009	0.008	−0.002
*APOE* (ε4 +)	−0.390^***^	−0.344^**^	−0.175	−0.154	0.101	0.004	−0.309^**^	−0.366^**^	0.094
AV45 PET SUVR	−3.048^***^	−2.827^***^	−0.504	−0.674	−1.368	−0.536	−1.265	−0.286	0.464
Plasma p‐tau181	−0.766^***^	−0.688^***^	−0.835^***^	−0.701^***^	−0.694^***^	−0.862^***^	−0.473^*^	−0.573^**^	0.766^***^

*Note*: β‐coefficients are derived from multivariate analysis using GLMs. Indicators of N were *z* scored before analysis. AV45 PET SUVR and plasma p‐tau181 levels were log‐transformed.

Abbreviations: *APOE*, apolipoprotein E; FDG‐PET, 18F‐fluorodeoxyglucose PET; GLMs, generalized linear models; HV, hippocampal volume; L, left; MRI, magnetic resonance imaging; N, neurodegeneration; NfL, neurofilament light chain; PET, positron emission tomography; p‐tau, phosphorylated tau; R, right; SUVR, standardized uptake value ratio.

* *P* < 0.05.

** *P* < 0.01.

*** *P* < 0.001.

### 3.4 Changes in indicators of N with the progression of AD pathology

**FIGURE 3:**
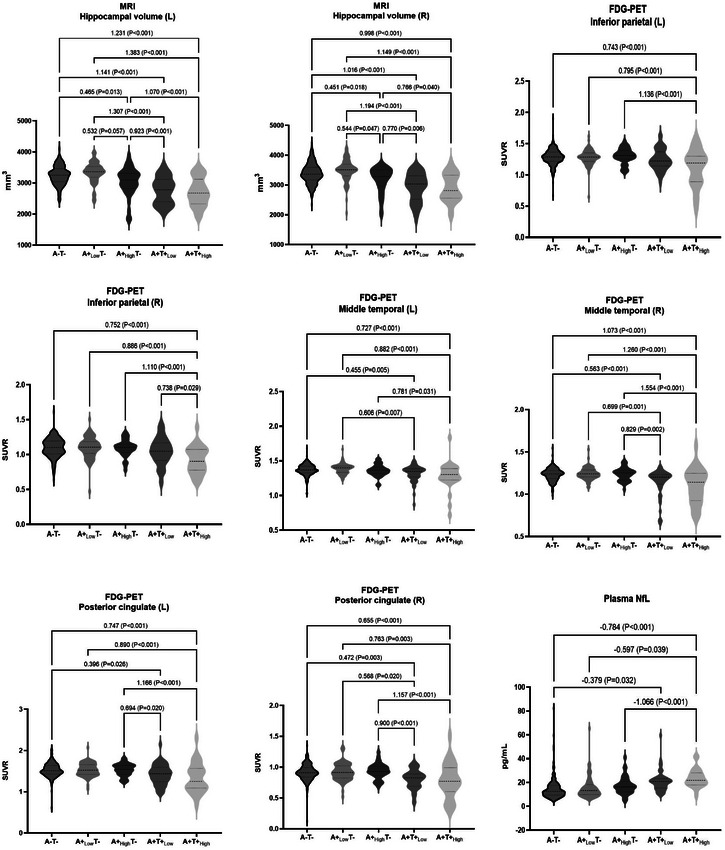
Levels of various indicators of neurodegeneration in the AD continuum. Group comparisons are performed using the ANCOVA with Bonferroni correction, and sex, age, education years, and *APOE* ε4 genotype are used as covariates. Effect sizes are reported as Cohen *d* to reflect differences between groups. A−, Aβ negative; T−, tau negative; A+_Low_, Aβ positive with lower‐third and middle‐third SUVR; A+_High_, Aβ positive with upper‐third SUVR; T+_Low_, tau positive with lower‐third and middle‐third plasma P‐tau181 level; T+_High_, tau positive with upper‐third plasma p‐tau181 level. Aβ, amyloid beta; AD, Alzheimer's disease; ANCOVA, analysis of covariance; *APOE*, apolipoprotein E; FDG, fluorodeoxyglucose; L, left; MRI, magnetic resonance imaging; PET, positron emission tomography; p‐tau, phosphorylated tau; R, right; SUVR, standardized uptake value ratio

Participants were divided into five groups: A−T− (*n* = 211), A+_Low_T− (*n* = 69), A+_High_T− (*n* = 34), A+T_Low_+ (*n* = 47), and A+T_High_+ (*n* = 25). Demographics revealed that A+_High_T− and A+T_Low_+ groups were older (68.56 and 68.04 years), the A+T_High_+ group had less education (9.34 years), and A+_High_T−, A+T_Low_+, and A+T_High_+ groups had more *APOE* ε4 carriers (44.1%, 46.8%, and 56%, respectively). ACE‐III‐CV scores declined progressively across A/T subgroups. Sex distribution was similar across groups. As illustrated in Figure 3, the HV‐L and HV‐R were significantly reduced in the A+T_Low_+ and A+T_high_+ subgroups relative to the A−T−, A+_Low_T‐, and A+_High_T− subgroups. Additionally, a reduction in HV‐L and HV‐R was also evident in the A+_High_T− subgroup compared to the A−T− and A+_Low_T− subgroups. A significant reduction of FDG PET SUVR in the IP‐L and IP‐R gyri was exclusively observed in the A+T+_High_ subgroup compared to the other subgroups. In the MT‐L and MT‐R gyri, FDG PET SUVRs were significantly lower in the A+T+_High_ subgroup than in the A−T−, A+_Low_T−, and A+_High_T− subgroups. Additionally, the A+T+_Low_ subgroup showed lower SUVRs compared to the A−T− and A+_Low_T− subgroups, and specifically, lower than the A+_High_T− subgroup in MT‐R. In the PC‐L and PC‐R gyri, FDG PET SUVRs were significantly lower in the A+T+_High_ subgroup than in the A−T−, A+_Low_T−, and A+_High_T− subgroups. Additionally, the A+T+_Low_ subgroup showed lower SUVRs compared to the A−T− and A+_High_T− subgroups, and specifically, lower than the A+_Low_T− subgroup in PC‐R. Plasma NfL levels were significantly higher in the A+T+_High_ subgroup than in the A−T−, A+_Low_T−, and A+_High_T− subgroups, and higher in the A+T+_Low_ subgroup compared to the A−T− subgroup. All the indicators of N had no significant difference between the A−T− and A+_Low_T− subgroups.

### 3.5 Associations between indicators of N and global cognitive decline

**FIGURE 4:**
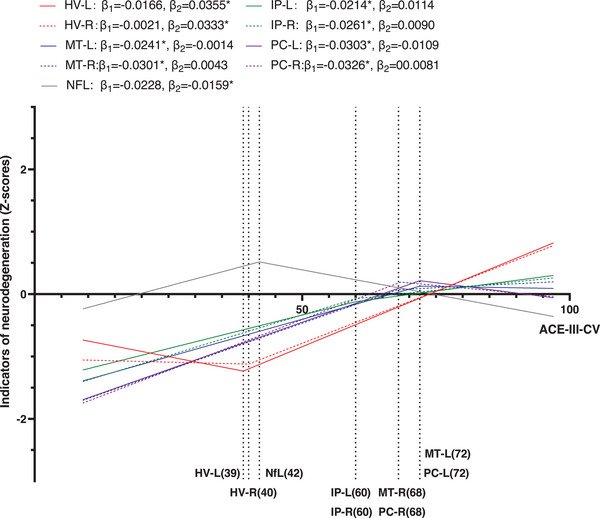
Variation characteristics of various indicators of N in relation to changes in global cognitive function. The lines represent trends based on segmental linear regression. Vertical dashed lines and corresponded ACE‐III‐CV scores were derived from the regression models best fit for various indicators of N. *β*_1_ and *β*_2_ indicate the regression slopes shown on the left and right of the vertical dashed lines. * Indicates statistically significant. ACE‐III‐CV, Chinese version of Addenbrooke's Cognitive Examination III; FDG, fluorodeoxyglucose; HV, hippocampal volume (magnetic resonance imaging); IP, inferior parietal (FDG PET); L, left; MT, middle temporal (FDG PET); N, neurodegeneration; NfL, neurofilament light chain (plasma); PC, posterior cingulate (FDG PET); PET, positron emission tomography; R, right

The associations between indicators of N and global cognitive decline were evaluated by segmental linear regression (Figure 4). The HV‐L and HV‐R exhibited significant correlations with the ACE‐III‐CV scores when the scores exceeded 39 and 40 (*β* = 0.0355 and 0.0333, respectively). However, no significant correlations were observed when the scores fell below these breakpoints. In comparison, FDG PET SUVRs showed significant correlations with the ACE‐III‐CV scores when the scores were < 60 (IP‐L, *β* = 0.0214; IP‐R, *β* = 0.0261), 68 (MT‐R, *β* = 0.0301; PC‐R, *β* = 0.0326), and 72 (MT‐L, *β* = 0.0241; PC‐L, *β* = 0.0303), while no significant correlations were observed when the scores exceeded the breakpoints. In addition, a significant correlation was observed between plasma NfL levels and ACE‐III‐CV scores when the score exceeded 42 (*β* = 0.0159), while no significant correlation was detected below this breakpoint.

### 3.6 Distribution of various N+ in different A/T statuses and effects on cognitive impairment

FDG+ was characterized by a low SUVR in any of the IP‐L or IP‐R, MT‐L or MT‐R, and PC‐L or PC‐R gyri, specifically falling below the 5th percentile of the distribution observed in CN individuals with Aβ negativity under the age of 60 within the C‐PAS cohort. Similarly, HV+ was defined by a reduction in HV‐L or HV‐R, while NfL+ was defined as having elevated plasma NfL levels exceeding the 95th percentile. Table 3 presents the distributions of various N+ types across different A/T stages and their associated impacts on cognitive impairment. The data are shown for both unadjusted models and models adjusted for age, sex, education years, and *APOE* ε4 genotype. In the A−T− subgroup, individuals with CI exhibited a significantly higher proportion of FDG (MT)+ and FDG (PC)+ compared to CN, and the presence of FDG (MT)+ and FDG (PC)+ was associated with an increased likelihood of cognitive impairment (odds ratio [OR] = 2.41 and 3.75 in unadjusted models, and OR = 2.48 and 4.84 in adjusted models, respectively). In the A–T+ subgroup, individuals with CI exhibited a significantly higher proportion of FDG (MT)+, which was associated with a markedly increased likelihood of cognitive impairment (OR = 7.00 in unadjusted model, and OR = 14.34 in adjusted model, respectively). In the A+T− subgroup, individuals with CI exhibited a significantly higher proportion of HV+ and NfL+. The HV+ and NfL+ were associated with an increased likelihood of CI in unadjusted models (OR = 3.90 and 2.73, respectively), while the significant associations disappeared in adjusted models. In the A+T+ subgroup, individuals with CI had a significantly higher proportion of HV+, FDG (IP)+, FDG (MT)+, and FDG (PC)+, but not NfL+. Furthermore, the presence of HV+, FDG (IP)+, FDG (MT)+, and FDG (PC)+ was associated with a significantly increased likelihood of cognitive impairment in unadjusted models (OR = 55.00, 17.30, 4.30, and 8.07, respectively). Upon adjusting for age, sex, education years, and *APOE* ε4 genotype, the associations with cognitive impairment remained significant for HV+ and FDG (IP)+ (OR = 71.15 and 11.22, respectively). However, the statistical significance was no longer observed for FDG (MT)+ and FDG (PC)+.

**TABLE 3:** Effects of FDG+, HV+, and NfL+ on cognitive impairment in different A/T statuses.

A/T/N	CN (*n* = 240)	CI (*n* = 176)	Model 1		Model 2	
			OR (95% c.i.)	*P*	OR (95% c.i.)	*P*
A−T−	146	65	N/A		N/A	
HV+	21 (14.4%)	16 (24.5%)	1.94 (0.93, 4.03)	0.074	1.82 (0.80, 4.15)	0.151
FDG+ (inferior parietal)	27 (18.5%)	11 (16.9%)	0.89 (0.41, 1.94)	0.784	0.666 (0.28, 1.56)	0.666
FDG+ (middle temporal)	20 (13.7%)	18 (27.7%)*	2.41 (1.17, 4.95)	0.016	2.48 (1.13, 5.46)	0.023
FDG+ (posterior cingulate)	6 (4.1%)	9 (13.8%)*	3.75 (1.27, 11.02)	0.016	4.84 (1.52, 15.40)	0.007
NfL+	17 (11.6%)	12 (18.5%)	1.71 (0.76, 3.84)	0.188	1.65 (0.67, 4.07)	0.269
A−T+	18	12	N/A		N/A	
HV+	3 (16.7%)	3 (25.0%)	1.66 (0.27, 10.09)	0.578	0.43 (0.02, 6.45)	0.544
FDG+ (inferior parietal)	4 (22.2%)	3 (25.0%)	1.16 (0.21, 6.48)	0.860	0.754 (0.08, 7.06)	0.805
FDG+ (middle temporal)	3 (16.7%)	7 (58.2%)*	7.00 (1.29, 37.90)	0.024	14.34(1.18,173.72)	0.036
FDG+ (posterior cingulate)	3 (16.7%)	1 (8.3%)	0.45 (0.04, 4.97)	0.518	0.267 (0.01, 4.60)	0.364
NfL+	5 (27.8%)	7 (58.3%)	3.64 (0.77, 17.03)	0.101	9.54 (0.80, 113.47)	0.074
A+T−	60	43	N/A		N/A	
HV+	6 (10.0%)	13 (30.2%)*	3.90 (1.34, 11.31)	0.012	2.37 (0.61, 9.09)	0.208
FDG+ (inferior parietal)	11 (18.3%)	8 (18.6%)	1.018 (0.37, 2.79)	0.972	0.79 (0.24, 2.52)	0.694
FDG+ (middle temporal)	10 (16.7%)	4 (9.3%)	0.28 (0.14, 1.75)	0.288	0.48 (0.11, 2.08)	0.330
FDG+ (posterior cingulate)	3 (5.0%)	4 (9.3%)	1.94 (0.41, 9.19)	0.399	0.554 (0.06, 4.82)	0.592
NfL+	9 (15.0%)	14 (32.6%)*	2.73 (1.05, 7.09)	0.039	3.37 (0.97, 11.69)	0.055
A+T+	16	56	N/A		N/A	
HV+	1 (6.3%)	44 (78.6%) *	55.00 (6.58, 459.35)	<0.001	71.15 (5.50, 919.44)	0.001
FDG+ (inferior parietal)	1 (6.3%)	30 (53.6%)*	17.30 (2.13, 140.10)	0.008	11.22 (1.29, 97.65)	0.028
FDG+ (middle temporal)	4 (25%)	33 (58.9%)*	4.30 (1.23, 15.03)	0.022	4.31 (0.97, 19.07)	0.054
FDG+ (posterior cingulate)	2 (12.5%)	30 (53.6%)*	8.07 (1.67, 38.89)	0.009	4.85 (0.78, 30.03)	0.090
NfL+	9 (56.3%)	33 (58.9%)	1.11 (0.36, 3.42)	0.848	1.20 (0.32, 4.42)	0.780

*Note*: The distributions of HV+, FDG+, and NfL+ are compared using chi‐square tests. ORs are derived from logistic regression models with cognitive impairment as outcome. Model 1 is not adjusted. Model 2 is adjusted for age, sex, education years, and *APOE* ε4 genotype. HV+, FDG+, and NfL+ were defined using threshold of the 5th percentile HVs, FDG PET SUVRs, and the 95th percentile plasma NfL levels based on cognitively normal young controls who are Aβ negative within the overall C‐PAS cohort, respectively.

Abbreviations: *APOE*, apolipoprotein E; c.i., confidence interval; CI, cognitively impaired; CN, cognitively normal; C‐PAS, Chinese Preclinical Alzheimer's Disease Study; FDG PET, 18F‐fluorodeoxyglucose positron emission tomography; HV, hippocampal volume; NfL, neurofilament light; ORs, odds ratios; SUVR, standardized uptake value ratio.

## 4 DISCUSSION

Our study found that HVs are more closely associated with plasma NfL levels in CN (Aβ+) individuals, while they are more strongly linked to FDG PET SUVRs in those with CI (Aβ+). Besides the effects via tau, an increased brain Aβ burden led to a reduction in HV. In contrast, reduced brain hypometabolism in the IP, MT, and PC gyri, as well as elevated plasma NfL levels, were primarily mediated by the effect of tau. During the progression of cognitive decline, HVs and plasma NfL levels were found to have significant correlations with cognitive function in the early to middle stages, whereas brain hypometabolism demonstrated a significant association with cognitive function in the middle to late stages. Furthermore, the presence of HV+ and FDG (IP)+ was associated with an increased risk of cognitive impairment in the A+T+ cohort. Conversely, FDG (MT)+ was linked to a heightened risk in both the A−T− and A‐T+ groups, while FDG (PC)+ was correlated with an elevated risk specifically in the A−T− group. The presence of NfL+ did not contribute to an increased risk in any of these populations.

In our GLM and mediation models, an increased Aβ burden exhibited a direct effect on hippocampal atrophy. Additionally, HV exhibited an earlier alteration in individuals with a high Aβ burden but who were T–. This is consistent with the literature reports that higher baseline brain Aβ was associated with more pronounced longitudinal hippocampal atrophy in CN populations.^20^ Conversely, previous studies identified a lack of correlation between brain Aβ deposition and hypometabolism.^21^, ^22^ This can be elucidated by our findings that brain hypometabolism in the IP, MT, and PC gyri were predominantly tau mediated, rather than directly associated with Aβ deposition. Additionally, while fluid neurodegenerative biomarkers are generally regarded as indicators of active neuronal damage and may be more sensitive to subtle neuronal dysfunction, our findings indicate that elevated plasma NfL levels were primarily associated with the exacerbation of tau pathology, rather than earlier Aβ deposition. All these data suggest that hippocampal atrophy may serve as a more sensitive indicator than brain hypometabolism and increased plasma NfL levels in the early neuropathological progression of AD. Our result seems to contradict the hypothetical model for the sequential emergence of N biomarkers proposed by Jack et al., in which abnormal FDG PET and fluid biomarkers precede detectable changes on MRI.^3^, ^23^ This may be attributed to the fact that the previous hypothesis was not directly derived from the effects of brain Aβ and tau on various indicators of N. Instead, it was primarily concluded from the observations related to these indicators in differentiating various cognitive stages, predicting cognitive decline, as well as their prevalence of abnormalities and rates of change during disease progression.^24^, ^25^, ^26^, ^27^ On the other hand, it should be noticed that akin to the accumulation of tau neurofibrillary tangles (NFT) in AD, cerebral atrophy typically initiates in the medial temporal regions and later extends to the isocortical cortices. As this study did not include cortical thickness measurements across various brain regions, certain limitations must be considered.

To be noted, CSF and PET classify a proportion of discordant Aβ results. Previous research has demonstrated that such discordance in Aβ results can reach up to 21% in the cognitively unimpaired population.^28^ Reduced CSF Aβ level may be more common and earlier than brain Aβ deposition detected by PET, while PET‐detected Aβ is more strongly associated with p‐tau.^28^, ^29^ In addition, the visual rating for determining florbetapir positivity tends to be more conservative than other PET approaches, in which visual rating exhibited lower sensitivity but higher specificity relative to the SUVR.^30^ Therefore, using CSF Aβ or SUVR for the qualitative assessment of Aβ positivity may result in the classification of certain individuals, initially identified as A−T− through visual rating, as A+T−. Because no significant differences were observed in various neurodegenerative markers between the A−T− and A+T− groups in our study, the impact of different qualitative methods on the progression of N markers associated with A/T changes may be limited. Similarly, the various approaches for defining tau can result in differing classifications. Tau PET tracks disease progression in a linear manner throughout the disease course.^31^ However, visual rating of tau PET demonstrated greater sensitivity but lower specificity compared to tau SUVR, and vice versa, particularly in the early stages of the disease. In contrast, CSF p‐tau may exhibit abnormalities prior to tau PET and maintains good specificity across different disease stages, although it may reach a relative plateau in the later stages of AD.^32^ At present, blood‐based p‐tau has been recognized as a biomarker with diagnostic utility for AD comparable to that of CSF p‐tau.^4^ Our study showed that, with the exception of FDG SUVR in IP‐R, there were no statistically significant differences in the levels of neurodegenerative indicators between the A+T_Low_+ and A+T_High_+ subgroups. This may be partially due to the fact that plasma p‐tau181 levels tend to plateau at Braak stage III and IV tau pathology, resulting in a diminished response to more advanced tau NFTs within the brain.^33^ A previous study demonstrated that p‐tau217 had a higher responsiveness to tau NFTs compared to p‐tau181.^34^ This suggests that using tau PET imaging or fluid p‐tau217 for the assessment of tau pathology in patients may yield a more precise representation of alterations in N markers during the advanced stages of AD.

In our study, HV and plasma NfL levels exhibited a significant correlation with global cognitive function during the early to middle stages of cognitive decline, while their correlations were no longer significant in the late stage of cognitive impairment. Similar findings were reported in previous studies, noting that while the annualized rate of hippocampal atrophy increases with worsening clinical symptoms, it becomes insignificant in rapidly progressing mild cognitive impairment and AD dementia populations.^35^, ^36^ In addition, changes in CSF tau levels show a weak link to cognitive impairment in late‐stage AD patients.^37^, ^38^ Therefore, we propose that hippocampal atrophy and fluid neurodegenerative biomarkers can indicate early cognitive impairment but may not align with the severity of cognitive decline in advanced cases. Conversely, our findings indicate that significant correlations between FDG PET SUVRs and global cognitive function were observed predominantly during the middle to late stages of cognitive decline rather than during the early stages. From this perspective, the superiority of FDG PET over structural MRI and fluid biomarkers in predicting short‐term cognitive decline, as reported in previous studies,^39^, ^40^ may be attributed to the temporal proximity of its detectable changes to the onset of significant cognitive impairment rather than to an inherently higher sensitivity to cognitive impairment.

It is important to acknowledge that neurodegenerative biomarkers may be influenced by a variety of confounding factors and exhibit abnormalities beyond A+ and T+ classifications.^41^, ^42^ Therefore, we conducted a more comprehensive analysis of their relationship with cognitive impairment across different A/T statuses. After controlling for variables such as sex, age, education years, and *APOE* ε4 genotype, the presence of HV+ and FDG (IP)+ significantly increased the risk of cognitive impairment within the A+T+ subgroup, with the risk associated with HV+ being particularly pronounced. Conversely, FDG (MT)+ is linked to a heightened probability of cognitive impairment in both the A−T− and A−T+ subgroups, whereas FDG (PC)+ is associated with an increased risk in the A−T− subgroup. These results implicate that hippocampal atrophy is especially pertinent for evaluating the risk of cognitive impairment in patients with a confirmed biological definition of AD, while more emphasis should be placed on the hypometabolism in the middle temporal and posterior cingulate regions in populations exhibiting non‐AD biological changes. In the A+T− population, none of the N+ results significantly increased the risk of cognitive impairment in the adjusted models. This appears inconsistent with previous reports that the presence of N+ markedly heightened the risk of cognitive impairment in individuals with A+.^43^, ^44^ We propose that this is due to our subdivision of A/T stages, which led to a reduced occurrence of tau pathology–mediated N+ results within the A+T− subgroup. Notably, despite a higher proportion, no significant difference in NfL+ was found between CN and CI individuals in the A+T+ group, and adding NfL+ to the A+T+ profile did not increase the risk of cognitive impairment. This may be because NfL+ appears early in A+T+ patients, preceding significant cognitive decline. This also aligns with the finding in familial AD that blood‐based NfL may rise during presymptomatic stages.^45^

Limitations should be noted in this study. First, our study did not assess all the neurodegenerative biomarkers typically derived from anatomical MRI, such as cortical thinning and ventricular enlargement, which limits comparisons to FDG PET and fluid biomarkers. Second, tau positivity in our study was defined using plasma p‐tau181 levels. Because elevated p‐tau can occur between Aβ deposition and NFT formation and tends to plateau in later tau pathology stages, there may be certain discrepancies between this definition and the analyses based on tau‐related NFTs in the brain. Third, the longitudinal relationship between the rate of change in neurodegenerative biomarkers, amyloid–tau pathology, and cognitive decline could not be determined due to the limited availability of follow‐up data. These issues will be further investigated in our future study.

In conclusion, hippocampal atrophy first relates to Aβ and later to tau, while brain hypometabolism and elevated plasma NfL are mainly driven by tau. HVs and plasma NfL levels correlate with cognitive decline during the early to middle stages, and to brain glucose metabolism in the middle to late stages. Furthermore, hippocampal atrophy significantly increases the risk of cognitive impairment in the A+T+ population, hypometabolism in different brain regions increases the risk of cognitive impairment across different A/T groups, while elevated plasma NfL levels demonstrate an insufficient correlation with cognitive impairment within the distinct A/T populations. Our findings represent a further investigation into the characteristics of AD‐related structural, metabolic, and fluid neurodegenerative biomarkers and lay the groundwork for the selection and interpretation of N biomarkers across diverse clinical contexts.

## CONFLICT OF INTEREST STATEMENT

The authors declare that they have no competing interests. Author disclosures are available in the supporting information.

## CONSENT STATEMENT

This study was approved by the ethics committee of Shanghai Sixth People's Hospital and performed in accordance with the ethical standards stated in the 1964 Declaration of Helsinki and subsequent amendments.

## Supporting information



Supporting Information
